# An Integrated Cervical Stabilization Exercise and Thai Self-Massage Approach for Managing Chronic Nonspecific Neck Pain in Young Adults: A Single-Blind Randomized Controlled Trial

**DOI:** 10.3390/ijerph23010111

**Published:** 2026-01-16

**Authors:** Vitsarut Buttagat, Warathon Mathong, Metira Kongchana, Kanittha Lowprasert, Sujittra Kluayhomthong, Pattanasin Areeudomwong

**Affiliations:** 1 Department of Physical Therapy, School of Integrative Medicine, Mae Fah Luang University, Chiang Rai 57100, Thailand; vitsarut.but@mfu.ac.th (V.B.); sujittra.klu@mfu.ac.th (S.K.); 2 Research Group on Smart Integrative Medicine and Technology Sustainability, Mae Fah Luang University, Chiang Rai 57100, Thailand

**Keywords:** cervical stabilization, Thai self-massage, chronic neck pain, neck disability

## Abstract

**Highlights:**

**Public health relevance—how does this work relate to a public health issue?**

- Chronic nonspecific neck pain represents a growing public health burden, particularly among young adults, contributing to reduced productivity, functional limitation, and early healthcare utilization.
- Scalable self-care interventions are increasingly important to address musculoskeletal pain in community and home settings where access to long-term supervised care is limited.

**Public health significance—why is this work of significance to public health?**

- This randomized controlled trial demonstrates that an integrative self-care program combining cervical stabilization exercises and Thai self-massage can reduce pain and disability in young adults with chronic nonspecific neck pain.
- The findings support the role of low-cost, non-pharmacological self-management strategies as part of population-level approaches to musculoskeletal health promotion.

**Public health implications—what are the key implications or messages for practitioners, policy makers and/or researchers in public health?**

- Public health practitioners may consider incorporating structured exercise and self-massage programs into community-based musculoskeletal health initiatives targeting young adults.
- Policymakers and researchers should further evaluate the long-term effectiveness, adherence, and scalability of integrative self-care interventions across diverse and higher-risk populations.

**Abstract:**

Background: Chronic nonspecific neck pain (CNNP) is a widespread musculoskeletal condition affecting individuals across all age groups. Although cervical stabilization exercises (CSE) and Thai self-massage have each demonstrated therapeutic potential, evidence regarding the effectiveness of the combined applications of CSE and Thai self-massage remains limited. This study aimed to investigate the effects of a combined program of CSE and Thai self-massage (CSTM) on pain intensity (PI), pressure pain threshold (PPT), and neck disability (ND) in young adults with CNNP. Methods: This single-blind randomized controlled trial was conducted at the Department of Physical Therapy, School of Integrative Medicine, Mae Fah Luang University, Thailand. Fifty young adults with CNNP were randomly assigned into two groups. The CSTM group performed CSE integrated with Thai self-massage, whereas the control group practiced stretching exercises exclusively. Both groups engaged in their respective programs three times per week for a duration of four weeks. PI, PPT, and ND were assessed at baseline, after four weeks (Week 4), and at a two-week follow-up (Week 6). Results: Both groups showed significant improvements in PI, PPT, and ND (*p* < 0.05), representing within-group comparisons, at Week 4 and Week 6. Furthermore, between-group comparisons at Week 4 and Week 6 indicated that the CSTM group achieved significantly greater improvements in PI and ND than the control group (*p* < 0.05). Conclusion: A four-week program combining CSE with Thai self-massage was effective in reducing pain intensity and neck disability in young adults with CNNP, with benefits maintained at short-term follow-up. Trial registration: Thai Clinical Trials Registry (TCTR20231102008), registered on 2 November 2023.

## 1. Introduction

Neck pain is one of the most prevalent musculoskeletal disorders worldwide and consistently ranks among the leading causes of disability, second only to low back pain in population-based studies [1,2,3]. Recent global estimates indicate that neck pain affects hundreds of millions of individuals across all age groups and remains a major contributor to years lived with disability worldwide [4,5]. Beyond individual suffering, neck pain imposes a substantial socio-economic burden, including increased work absenteeism, reduced productivity, and rising healthcare expenditures [2,3,6], with its global impact well documented in epidemiological research [7]. Clinically, neck pain is commonly classified according to symptom duration as acute (<3 weeks), subacute (4–12 weeks), and chronic (>12 weeks) [8].

Nonspecific neck pain (NNP) is defined as pain localized to the cervical region, with or without radiation to the upper limbs, that cannot be attributed to a specific underlying disease or structural lesion [3,9]. This condition is strongly associated with modifiable risk factors, including sustained poor posture, prolonged screen time, repetitive movement, and insufficient ergonomic support [10,11]. Biomechanically, NNP is often linked to muscle–joint imbalance, reduced deep cervical flexor activation, increased superficial muscle overactivity, and impaired proprioceptive control [12,13,14]. Physiological alterations in individuals with chronic NNP (CNNP) include reduced isometric strength and endurance of cervical flexor and extensor muscles compared with healthy individuals [15]. Moreover, disturbances in sensorimotor control and altered postural stability have been documented, which may perpetuate pain and functional limitation over time [16,17,18]. These findings highlight that CNNP is not merely a localized pain condition but a multifactorial problem involving neuromuscular, biomechanical, and behavioral components.

Cervical stabilization exercises (CSEs) specifically target the deep cervical muscles, aiming to restore neuromuscular coordination between the deep and superficial neck musculature [19,20,21]. This approach has become increasingly recognized in the management of NNP, as it helps reduce mechanical strain, muscular fatigue, and nociceptive input [20,22,23,24]. Previous studies have demonstrated that exercises focusing on deep cervical muscle activation can improve neuromotor control and decrease pain and disability [25,26,27]. More recently, Olofinbiyi et al. [28] reported that CSE effectively reduced pain intensity and improved neck-related function in individuals with CNNP.

Thai massage, a traditional therapeutic practice with historical and cultural significance, is supported by contemporary evidence demonstrating its effectiveness in reducing musculoskeletal pain, promoting relaxation, improving circulation, and enhancing psychological well-being [29,30,31,32,33]. Specifically targeting the neck region, Thai massage has also been reported as an effective intervention for relieving pain and improving function in individuals with chronic neck pain [34].

Several studies have demonstrated that combining manual therapy with exercise produces greater clinical benefits than exercise alone in individuals with CNNP. Randomized controlled trials have shown that integrated programs lead to significantly larger reductions in PI and disability, as well as improvements in cervical motion and quality of life compared with exercise-only approaches [21,35]. A recent systematic review and meta-analysis further confirmed these findings, reporting moderate-to-large, pooled effect sizes favoring the combined intervention for both pain and disability outcomes [36]. These results support the synergistic role of manual therapy and exercise in enhancing neuromuscular function and alleviating symptoms in patients with chronic neck pain.

Recent healthcare trends emphasize the importance of self-care as an effective and sustainable strategy for managing chronic musculoskeletal disorders [37,38,39]. Self-care refers to individuals’ ability to maintain health, prevent illness, and manage symptoms with or without support from healthcare providers [39]. Research has shown that structured home-based rehabilitation and self-management programs can improve physical and psychological outcomes, enhance treatment adherence, and reduce healthcare costs compared with conventional hospital-based care [40,41]. Moreover, self-care interventions empower individuals to take an active role in their own health, promoting independence and long-term behavioral change [42]. To optimize therapeutic benefit, previous studies emphasize that supervision by a qualified clinician throughout the exercise or manual therapy program helps ensure correct technique, prevent compensatory patterns, and reduce the risk of symptom exacerbation [43,44,45].

Given these benefits, integrating self-administered interventions such as CSE and Thai self-massage may not only alleviate pain and disability but also reduce healthcare dependency while enhancing overall quality of life. However, evidence supporting the effectiveness of a combined program of CSE and Thai self-massage (CSTM) for CNNP remains limited. Therefore, the present study aimed to examine the effects of an integrative self-care program combining CSE with self-administered Thai massage on pain intensity (PI), pressure pain threshold (PPT), and neck disability (ND) in individuals with CNNP.

## 2. Materials and Methods

### 2.1. Study Design and Setting

This single-blind randomized controlled trial compared the effects of a CSTM program with a control condition involving stretching exercises in patients with CNNP. The study was conducted at the Department of Physical Therapy, School of Integrative Medicine, Mae Fah Luang University, Chiang Rai, Thailand, between November 2023 and June 2024. Prior to enrollment, participants were randomly assigned to either the CSTM or control group in a 1:1 ratio using permuted-block randomization with block sizes of two, four, and six generated by STATA version 10 (StataCorp LP, College Station, TX, USA). The randomization sequence was prepared by a research assistant who was not involved in participant recruitment, intervention delivery, or outcome assessment. Group allocations were concealed in sequentially numbered, opaque, sealed envelopes to maintain allocation confidentiality. Outcome measures, including PI, PPT, and the Neck Disability Index (NDI), were assessed at baseline, after the four-week intervention period, and at a two-week follow-up. The study protocol was approved by the Mae Fah Luang University Ethics Committee on Human Research on 23 August 2023 (EC 23094-25) and prospectively registered with the Thai Clinical Trials Registry on 2 November 2023 (TCTR20231102008). Written informed consent was obtained from all participants before enrollment. This trial was designed and reported in accordance with the CONSORT 2010 Statement to ensure methodological rigor and transparency.

### 2.2. Participants

Patients with CNNP were recruited from Chiang Rai Province, Thailand, via social media campaigns and advertisement posters. Inclusion criteria were: male or female aged 18 to 60 years; history of neck pain persisting for at least 12 weeks; and the ability to understand and communicate effectively in Thai to ensure compliance with study procedures and assessments. Exclusion criteria included neck pain attributable to trauma, disk herniation, whiplash injury, congenital spinal deformity, spinal stenosis, neoplasm, inflammatory rheumatic disease, neurological disorders, or other identifiable pathologies. Additionally, individuals presenting contraindications to Thai self-massage or exercise, such as fever, acute muscle or tendon injury, uncontrolled hypertension, bone fractures, or joint dislocations, were also excluded.

### 2.3. Sample Size Calculation

The required sample size for this study was determined using an a priori power analysis in G*Power software (Version 3.1.9.6) (Heinrich Heine University Düsseldorf, Düsseldorf, Germany), utilizing the ‘Means: Difference between two independent means’ statistical test. Based on an internal pilot study (*n* = 5 per group) comparing the CSTM and stretching programs, the primary outcome (Pain-Visual Analog Scale) data showed a mean of 2.8 (SD = 0.8) for the CSTM group and 1.4 (SD = 1.8) for the stretching group. These values resulted in an estimated Effect Size (Cohen’s d) of 1.01. With a two-tailed significance level of 0.05 and a statistical power of 0.90, the analysis required a minimum of 22 participants per group. To account for an anticipated 10% dropout rate, 25 participants were ultimately recruited for each group.

### 2.4. Outcome Measures

All participants were assessed by blinded investigators at three time points: (1) before the intervention (baseline), (2) after completion of the 4-week program (Week 4), and (3) two weeks after the intervention (Week 6). The assessors were blinded to both group allocation and assessment timepoints throughout the study to minimize assessment bias.

#### 2.4.1. Primary Outcome

##### Pain Intensity (PI)—Visual Analog Scale (VAS)

PI was assessed using a 10 cm horizontal VAS, ranging from “no pain” (0) to “worst pain imaginable”. Participants were instructed to rate their current neck pain intensity at the time of each assessment session. The distance in centimeters from the “no pain” anchor to the participant’s mark was measured to obtain the VAS score, with higher scores indicating greater pain. The VAS demonstrates good reliability for musculoskeletal pain assessment, with reported Intraclass Correlation Coefficient (ICC) values ranging from 0.71 to 0.94 and a minimal clinically important difference (MCID) of approximately 1.2 cm in neck pain populations [46,47].

#### 2.4.2. Secondary Outcomes

##### Pressure Pain Threshold (PPT)

PPT was assessed using a digital pressure algometer (OE-220, ITO Co., Ltd., Tokyo, Japan). Participants were seated upright with their back supported, elbows flexed at 90°, forearms resting on their thighs, and feet placed flat on the floor. Pressure was applied perpendicularly to the skin over the most tender point of the upper trapezius muscle at a constant rate until the participant perceived the first sensation of pain and pressed the stop button on the device to indicate this point. To ensure measurement consistency across all time points, the exact location of the most tender point was mapped using a transparent plastic sheet referenced against anatomical landmarks (e.g., the C7 spinous process and the acromion). Only the most symptomatic side was assessed in this study, as the primary objective was to evaluate the localized treatment effect on the specific myofascial trigger point identified. The algometer reading (kg/cm^2^) at that moment was recorded as the PPT value. Three measurements were obtained at 30 s intervals, and the mean of these readings was used for analysis to enhance measurement reliability [48,49]. PPT measurement over the cervical region demonstrates excellent intra-rater reliability (ICC = 0.87–0.90), and the MCID is reported to be 0.63 kg/cm^2^ [50].

##### Neck Disability Index (NDI)

The Thai-adapted version of the Neck Disability Index (NDI) was employed to determine the level of functional limitation associated with neck pain. The instrument consists of ten sections covering daily activities such as self-care, lifting, reading, and driving. Each item is rated on a six-point ordinal scale, where 0 represents no disability and 5 indicates complete disability. The total score is used to categorize the severity of neck-related disability [51]. These categories include No Disability (0–4 points), Mild Disability (5–14 points), Moderate Disability (15–24 points), Severe Disability (25–34 points), and Complete or Very Severe Disability (35–50 points). The NDI has shown strong test–retest reliability, with ICCs reported between 0.74 and 0.91 [49]. The MCID for the NDI is also reported to be 3 to 5 points [52].

##### Adverse Events

Adverse events were monitored throughout the intervention period. After each treatment session, participants were asked whether they experienced any discomfort, unusual symptoms, or adverse reactions related to the intervention. All responses were documented by the same assessor who conducted the outcome measurements and subsequently reviewed by the research team to ensure participant safety.

### 2.5. Interventions

The study included two intervention groups: the CSTM group and the control group, which performed stretching exercises only. All interventions were delivered onsite at the Physical Therapy Clinic, School of Integrative Medicine, Mae Fah Luang University, and each session was fully supervised by a licensed physical therapist with more than 15 years of clinical experience to ensure correct technique, promote adherence, and maintain participant safety. Intervention adherence was monitored using session attendance records for all supervised sessions. The exercise intervention was reported in accordance with the Consensus on Exercise Reporting Template (CERT) to enhance transparency and reproducibility.

#### 2.5.1. Cervical Stabilization Exercises and Thai Self-Massage Group (CSTM)

Participants in the CSTM group performed a series of CSE followed by self-administered Thai self-massage three times per week, 40 min per session, for four consecutive weeks. Each session comprised 30 min of exercise and 5 min of self-massage, separated by a 5 min rest period.

##### Cervical Stabilization Exercises (CSE)

**Figure 1:**
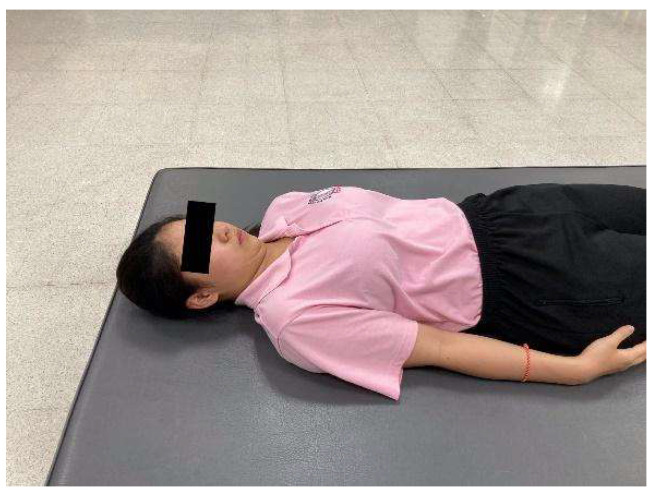
Chin tuck.

These exercises were designed to activate the deep cervical muscles, enhance postural control, and improve neuromuscular coordination. Exercise progression followed a standardized sequence from low-load deep muscle activation (chin tuck, towel-supported nodding) to higher-load stabilization tasks (head lift, elastic-band resistance, isometric ball press). The therapist continuously monitored for common compensatory patterns, including excessive sternocleidomastoid activation, jaw clenching, shoulder elevation, or breath-holding. Corrections were given immediately to maintain proper form and ensure selective activation of deep neck flexors. The exercise protocol was standardized and did not include individualized progression. All participants performed the same exercises with identical dosage and sequence throughout the intervention period to ensure protocol consistency and reproducibility. The protocol consisted of the following movements:

Chin tuck—Participants lay supine with the spine in a neutral position and gently drew the chin inward toward the throat, as if making a “double chin,” while keeping the head in contact with the surface (Figure 1). The position was held for 5 s and repeated 10 times.

**Figure 2:**
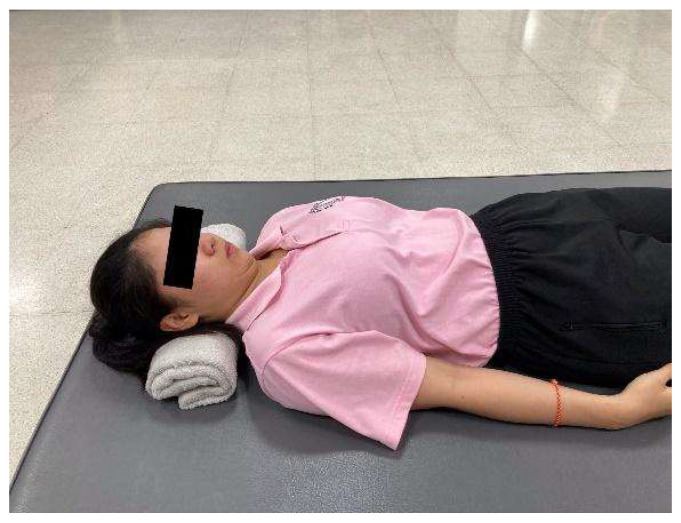
Towel-supported nodding.

Towel-supported nodding—A small, rolled towel was placed under the occiput to provide light support. Participants performed a gentle nodding motion (“yes” gesture) by pressing the back of the head slightly downward into the towel without lifting the head off the surface using minimal visible effort (Figure 2). This gentle pressure was held for 5 s before relaxing. The exercise was repeated 10 times.

**Figure 3:**
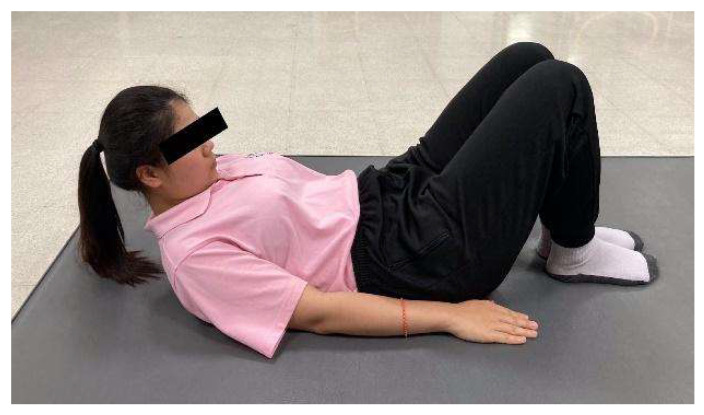
Head lift with chin tuck.

Head lift with chin tuck—While maintaining a chin tuck, participants lifted the head approximately 3 cm from the surface, keeping the neck aligned and eyes focused on the ceiling. The contraction was held for 5 s before slowly returning to the starting position (Figure 3). This movement was repeated 10 times.

**Figure 4:**
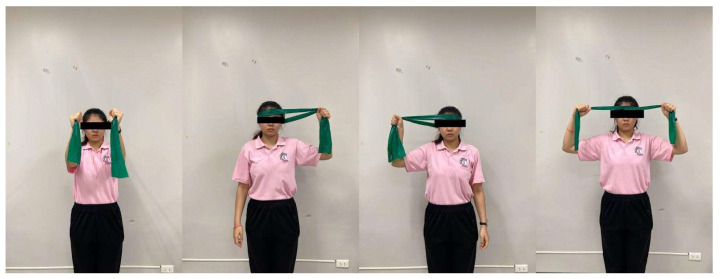
Elastic-band resistance training.

Elastic-band resistance training—Participants stood upright with a neutral spine and performed isometric contractions of the neck muscles against resistance from a Theraband in four directions (flexion, extension, left, and right lateral flexion) (Figure 4). Each contraction was held for 10 s and repeated 10 times per direction.

**Figure 5:**
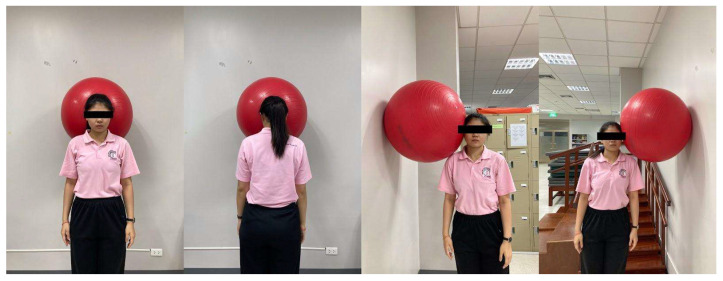
Isometric ball press.

Isometric ball press—In a standing position, participants placed a soft ball between the back of the head and a wall, gently pressing in four directions (anterior, posterior, left, right) while maintaining neutral alignment (Figure 5). Each press was held for 10 s and repeated 10 times in each direction.

Following the completion of the exercise protocol, participants were instructed to rest for five minutes prior to commencing the self-massage component.

##### Thai Self-Massage

Before initiating the self-massage protocol, participants received standardized instruction and demonstration from the supervising physical therapist. Correct technique and pressure application were verified through supervised practice prior to program implementation.

**Figure 6:**
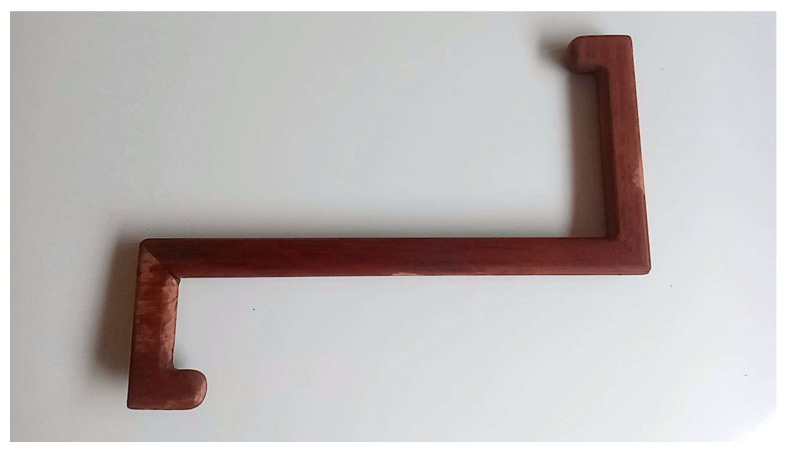
Wooden massage tool.

**Figure 7:**
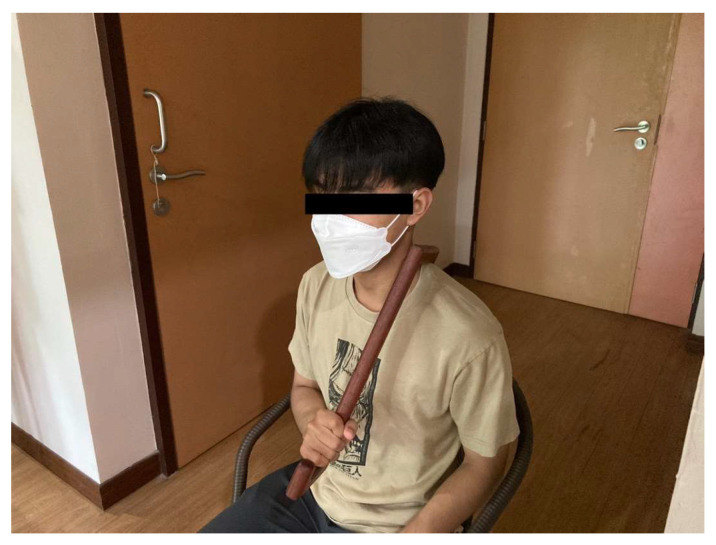
Thai Self-Massage performed with the wooden massage tool.

**Figure 8:**
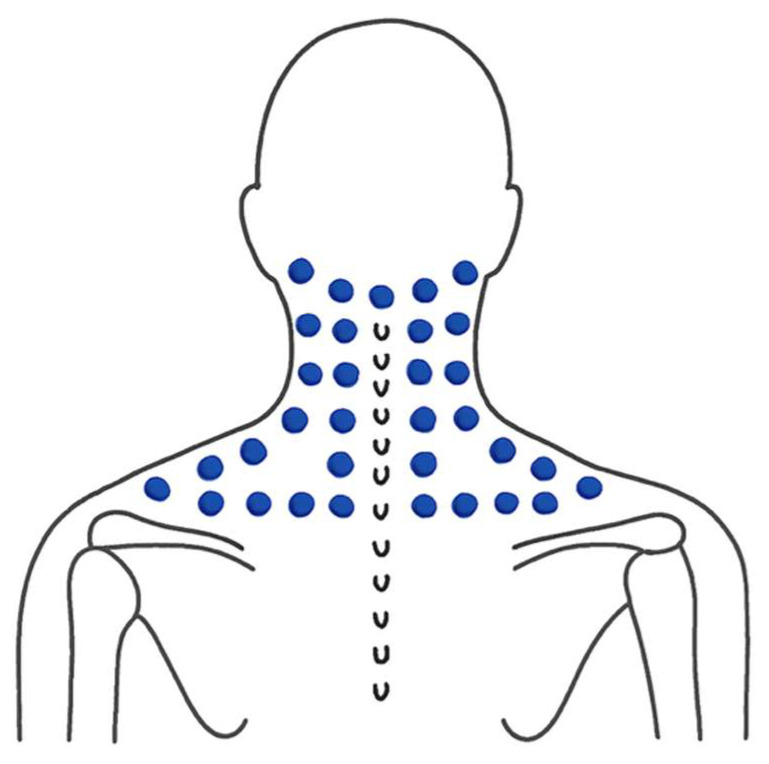
Specific lines for applying self-administered Thai massage on the upper back and neck.

Participants performed self-massage using a custom-designed wooden massage tool created by the research team (Figure 6). In a seated position (Figure 7), they held the handle and applied steady, rhythmic pressure along specific massage lines (Figure 8), following the traditional Thai massage principles outlined by Eungpinichpong [53]. The pressure was gradually increased until a mild sensation approaching the individual’s pressure pain threshold was felt. At each site, the pressure was sustained for five seconds before being released, and the procedure was repeated three times along each massage line.

#### 2.5.2. Control Group

**Figure 9:**
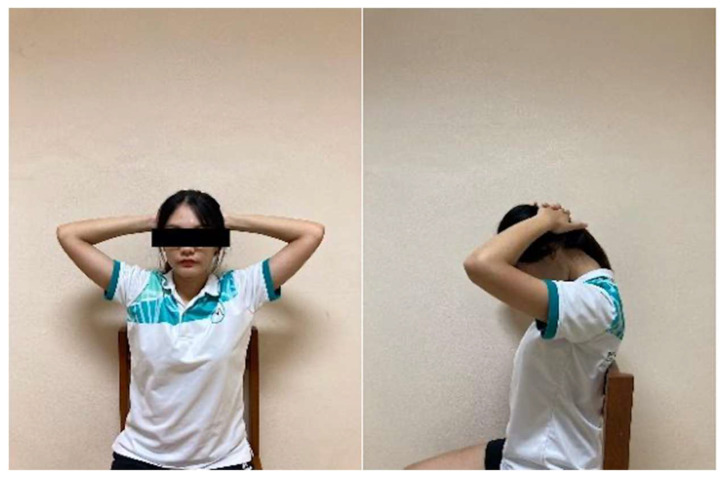
Neck flexion stretch.

Participants in the control group performed a series of five stretching exercises targeting the cervical region. Each stretch was performed slowly and steadily until a gentle tension, but not pain, was felt, and was held for 15 s followed by a 5 s rest interval before returning to the starting position. The total session duration was approximately 40 min, matching the treatment dose of the CSTM group to ensure equal time exposure. This duration accounted for the stretching repetitions, rest periods, and the necessary time for postural alignment and transitions between exercises. The same experienced physical therapist who supervised the CSTM group also supervised this group to minimize inter-provider variability. The specific procedures for the five cervical stretching exercises are presented below:

Neck Flexion Stretch: In a seated position, participants clasped their hands behind the head and gently bent the neck forward, bringing the chin toward the chest until a comfortable stretch was felt at the back of the neck (Figure 9). The stretch was held for 15 s and repeated 10 times with 5 s rests between repetitions.

**Figure 10:**
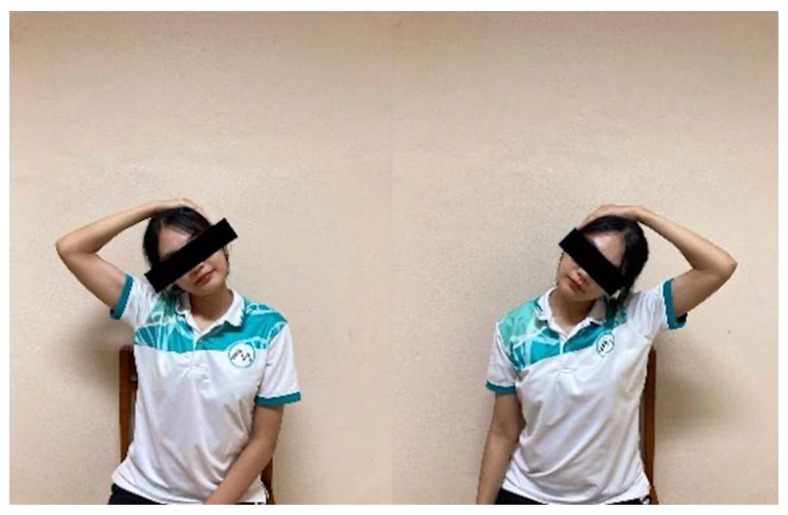
Neck lateral flexion stretch.

Neck Lateral Flexion Stretch: While seated upright, participants grasped the edge of the chair with one hand to stabilize the shoulder on that side. With the opposite hand, they gently tilted the head toward the opposite shoulder until a mild stretch was perceived along the side of the neck (Figure 10). The position was maintained for 15 s and repeated 10 times on each side with 5 s rests.

**Figure 11:**
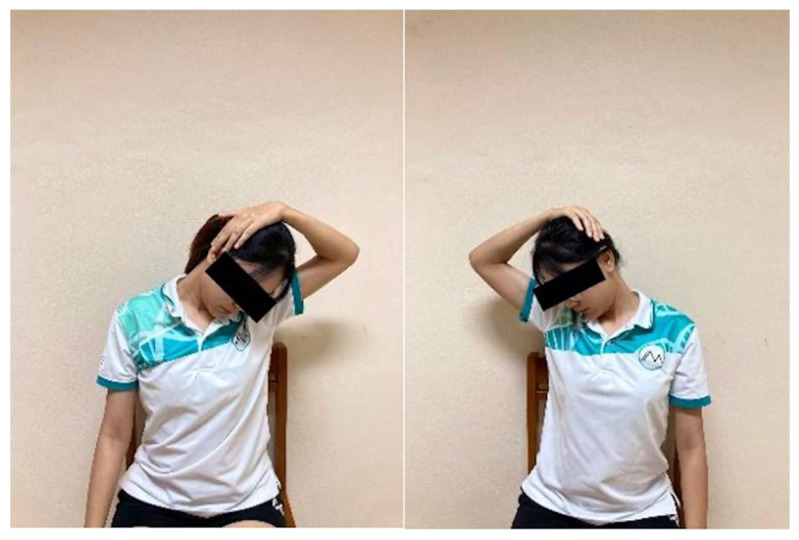
Neck Flexion with Rotation Stretch.

Neck Flexion with Rotation Stretch: Participants slowly bent the head forward and rotated it toward one side, as if looking toward the knee. Using one hand placed gently on the back of the head, they applied light pressure to increase the stretch (Figure 11). The position was held for 15 s and repeated 10 times on each side with 5 s rests.

**Figure 12:**
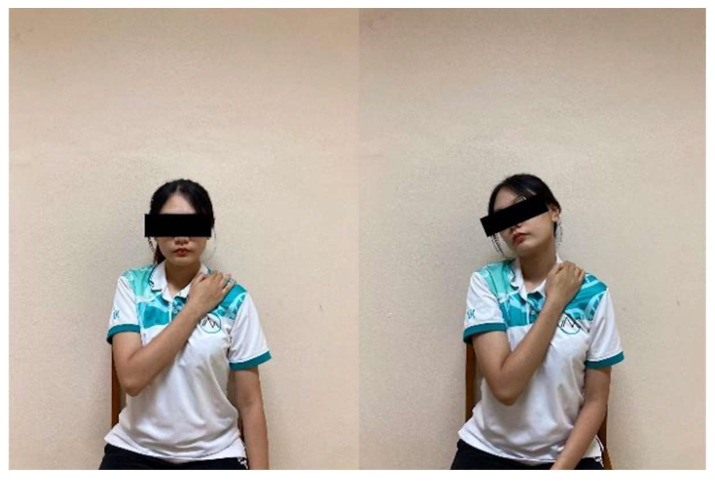
Anterolateral Neck Stretch.

Anterolateral Neck Stretch: In a seated position, participants placed one hand over the opposite clavicle to stabilize the shoulder. They then extended and side-bent the head away from the stabilized shoulder until a gentle stretch was felt at the front and side of the neck (Figure 12). Each stretch was held for 15 s and repeated 10 times on each side with 5 s rests.

**Figure 13:**
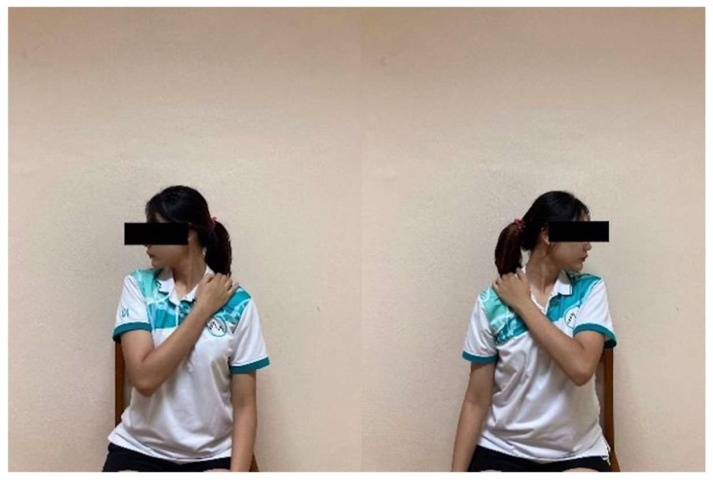
Neck Rotation Stretch.

Neck Rotation Stretch: While sitting upright, participants placed one hand on the opposite shoulder to prevent movement and gently rotated the head to the other side until a comfortable stretch was felt (Figure 13). The stretch was maintained for 15 s and repeated 10 times per side with 5 s rests.

### 2.6. Data Analysis

Data were analyzed using the Statistical Package for the Social Sciences (SPSS) version 20.0 (IBM Corp., Armonk, NY, USA). Descriptive statistics, including mean, standard deviation, and percentage, were used to summarize participant characteristics, and the Shapiro–Wilk test was applied to assess the normality of data distribution. All primary analyses adhered to the intention-to-treat (ITT) principle, with missing data managed via the Last Observation Carried Forward (LOCF) approach. Within-group differences over time were examined using repeated-measures analysis of variance (ANOVA), followed by Bonferroni-adjusted pairwise comparisons for post hoc analysis. Between-group comparisons of post-intervention outcomes were conducted using analysis of covariance (ANCOVA), controlling for baseline scores, body weight, and height to adjust for potential anthropometric influences on cervical biomechanical loading and individual variability in pain perception and functional performance. Statistical significance was established at an alpha level of (*p* < 0.05).

## 3. Results

**Figure 14:**
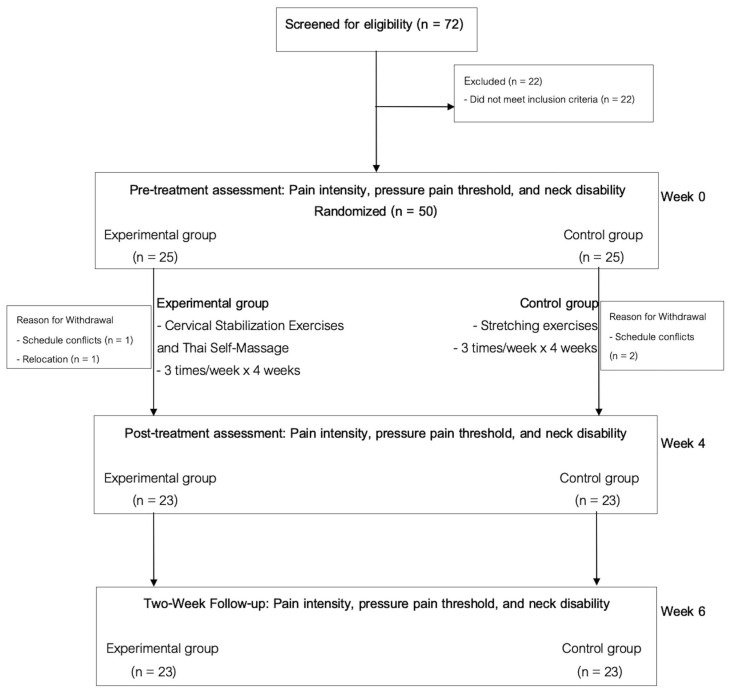
The CONSORT flow diagram.

A diagram illustrating the flow of participants throughout the trial is presented in Figure 14. A total of 72 individuals were screened for eligibility, of whom 50 met the inclusion criteria and provided informed consent to participate. Participants were randomly allocated to either the CSTM group (*n* = 25) or the control group (*n* = 25). Before receiving the first treatment session, two participants from the CSTM group withdrew due to schedule conflicts (*n* = 1) and relocation (*n* = 1), while two participants from the control group withdrew because of schedule conflicts. These reasons were unrelated to the intervention. Baseline demographic characteristics of the participants are summarized in Table 1. Participants in both groups completed all scheduled intervention sessions (12 of 12 sessions), corresponding to a 100% adherence rate.

No adverse events or treatment-related complications were reported by participants in either the CSTM or control group during the entire intervention period.

### Effect of the Interventions

All outcome measures are summarized in Table 2 and Table 3. Both groups showed significant improvements in PI, PPT, and NDI scores following the final treatment session and at the two-week follow-up (*p* < 0.05) (Table 2). Between-group comparisons of adjusted post-intervention values demonstrated significantly greater reductions in PI and NDI in the CSTM group compared with the control group (*p* < 0.05). Although the CSTM group also exhibited a greater mean increase in PPT compared with the control group, this difference did not reach statistical significance (*p* > 0.05) (Table 3).

## 4. Discussion

This study is the first to evaluate the clinical effectiveness of a CSTM program as a self-administered intervention for individuals with CNNP. The findings demonstrated that a 4-week CSTM program significantly reduced neck PI and disability while increasing the PPT from baseline. Furthermore, the CSTM program yielded greater improvements in pain and functional outcomes compared with stretching exercises alone. These results indicate that CSTM is an effective, safe, and practical self-management approach for individuals with CNNP.

When interpreting the findings, both statistical significance and clinical relevance should be considered. The MCID has been reported to be approximately 1.2 cm for pain intensity measured by the VAS, 3–5 points for the NDI, and 0.63 kg/cm^2^ for PPT [43,44,47,48]. Within the CSTM group, all outcome measures demonstrated statistically significant improvements, and the magnitude of change in PI, NDI, and PPT exceeded their respective MCID values, indicating clinically meaningful improvements at the individual level. In contrast, although between-group differences in PI and NDI favored the CSTM group and reached statistical significance, these differences did not exceed established MCID thresholds, while no significant between-group difference was observed for PPT. Clinically, this suggests that the additional benefit of CSTM over stretching alone was modest at the group level.

It is also important to interpret these findings in light of the study design. The control group received an active intervention consisting of supervised stretching exercises rather than a placebo or usual-care condition. The significant within-group improvements observed in the control group underscore the therapeutic value of stretching alone for individuals with CNNP. Therefore, the superior outcomes observed in the CSTM group should be interpreted as reflecting the added benefit of a more comprehensive, multimodal self-care program rather than the isolated, specific effects of cervical stabilization exercises or Thai self-massage. While the use of an active comparator enhances clinical relevance, it limits causal inferences regarding the contribution of individual treatment components.

The limited between-group clinical impact may be partly explained by the relatively mild baseline symptom severity of the participants, which inherently constrains the potential for large clinically meaningful differences. In addition, stabilization-based interventions for chronic neck pain are commonly delivered over longer durations (6–8 weeks) to achieve clinically detectable between-group effects [28,54], and the four-week intervention period in the present study may have been insufficient to fully capture the intervention’s comparative clinical benefit. It is also worth noting that the effect size used for the sample size calculation (d = 1.01), derived from preliminary data, was larger than the between-group effect sizes observed in the present study. This discrepancy likely reflects the tendency of small pilot samples to overestimate treatment effects. Therefore, although statistically significant differences were observed, the magnitude of between-group effects should be interpreted with appropriate caution.

Importantly, as a self-care–based intervention, patients can independently perform the CSTM program once adequately trained, facilitating ongoing symptom management beyond the clinical environment. Moreover, the wooden massage tool used in this program is inexpensive, simple to construct, and widely accessible, making the approach cost-effective and suitable for routine home implementation. Consequently, the CSTM program may serve as an accessible adjunct or alternative to conventional clinical care, promoting patient empowerment and reducing both healthcare and travel costs.

No adverse events were reported during the intervention period, confirming that both the CSTM and stretching exercise programs were safe and well tolerated. This finding further supports the integration of self-administered interventions, such as Thai self-massage and CSE, into conservative management strategies for individuals with CNNP.

These findings align with previous research demonstrating the benefits of multimodal exercise and manual therapy approaches for patients with CNNP. Sun et al. [55] reported that combining cervical and scapular stabilization exercises with self-mobilization training effectively reduced pain and disability while improving deep neck flexor strength, endurance, and cervical mobility. Similarly, Ghodrati et al. [56] found that integrating soft tissue release, muscle energy techniques, and cervical exercises produced significant reductions in PI and neck-related disability. Domingues et al. [35] also observed that participants receiving a combined program of manual therapy and cervical exercise exhibited greater improvements in pain, disability, and perceived recovery compared with those receiving usual care. In addition, Buttagat et al. [57] demonstrated that the integration of traditional Thai massage, scapular stabilization exercise, and chest mobilization improved forward head angle and cervical motion in individuals with forward head posture. Collectively, these findings support the therapeutic value of combining exercise with manual or self-administered techniques to address multiple impairments associated with CNNP.

Although the CSTM group demonstrated significant within-group improvement in PPT, the between-group comparison did not reveal a statistically greater change compared with the stretching group after the intervention period. It is possible that the four-week duration of the program was insufficient to produce measurable between-group differences in PPT.

The therapeutic effects observed in the present study may be hypothetically explained by complementary neuromuscular and myofascial mechanisms associated with the individual components of the CSTM program, as suggested by previous literature. CSE have been shown to activate the deep cervical flexor and extensor muscles, potentially enhancing segmental control, proprioceptive acuity, and postural stability of the cervical spine [16,25,27,35,55,58,59]. Improved coordination between deep and superficial cervical musculature has been proposed to reduce excessive loading on passive spinal structures and attenuate nociceptive input from muscle and joint tissues [25,35,58]. Similarly, evidence from prior studies indicates that Thai massage may alleviate symptoms through mechanical and neurophysiological pathways, such as increased local blood circulation, stimulation of mechanoreceptors, and modulation of pain perception consistent with gate-control theory [53,60,61,62,63]. The rhythmic pressure and sustained compression applied along Thai massage lines have also been suggested to facilitate myofascial release, thereby reducing soft-tissue stiffness and improving movement efficiency [34,53,60,61,62,63]. When applied sequentially, these two interventions may theoretically exert complementary effects, whereby exercise enhances neuromuscular control while self-massage addresses myofascial restrictions. However, it should be emphasized that these mechanisms were not directly measured in the present study and are inferred from existing literature examining each intervention separately.

This study has several limitations that should be considered when interpreting the results and planning future research. First, the follow-up period was relatively short, with the final assessment conducted only two weeks after the treatment program was completed. This limited timeframe prevents conclusions regarding the long-term effectiveness and sustainability of treatment benefits. Future studies should incorporate longer follow-up periods to confirm the durability of the CSTM intervention. Second, the homogenous sample of young adults limits external validity, and the findings may not be generalizable to older or more clinically diverse populations. Third, the four-week intervention duration may have constrained the ability to detect larger or more robust between-group differences. Previous studies suggest that longer intervention periods may be required to achieve more pronounced changes in certain outcomes, such as pressure pain threshold. Future trials should examine the effects of extended treatment durations. Fourth, the sample size calculation was based on effect size estimates derived from a small pilot study. Although pilot data are commonly used for preliminary sample size estimation, the limited sample size may have led to an overestimation of the true treatment effect due to increased variability. This potential inflation of effect size should be considered when interpreting the magnitude of between-group differences. Larger pilot studies or adaptive sample size re-estimation approaches may help generate more robust effect size estimates in future research. Fifth, the lack of blinding of both participants and treating therapists represents a major methodological limitation of this study. Owing to the distinct nature of the interventions, which involved different movement patterns and tactile components, blinding was not feasible. This design may have introduced expectation and performance bias, particularly affecting subjective, self-reported outcomes such as pain intensity and neck disability. Although outcome assessments were conducted by blinded assessors to minimize detection bias, the magnitude of expectancy-related effects cannot be fully determined and may have contributed to the observed between-group differences. Sixth, intervention fidelity outside the supervised sessions was not formally assessed. Although adherence to all on-site sessions was fully monitored, data on the frequency or correctness of unsupervised home practice were not collected. As self-care is a core component of the CSTM program, this limits interpretation regarding real-world adherence and may have influenced treatment outcomes. Future studies should include standardized monitoring of home-based practice. Finally, the use of an active control condition constitutes an additional limitation. While this approach enhances clinical relevance, it limits the ability to isolate the specific effects of cervical stabilization exercises or Thai self-massage from non-specific or additive effects associated with receiving a more comprehensive, multimodal intervention. Accordingly, the findings should be interpreted as reflecting the comparative effectiveness of a combined intervention approach relative to a single-modality active intervention, rather than evidence of isolated treatment-specific efficacy.

### Clinical Implications

The CSTM program provides a feasible and cost-effective option for managing CNNP within both clinical and community settings. Given its self-administered nature, patients can continue the program independently after appropriate instruction, reducing reliance on clinical supervision. Physical therapists and other rehabilitation professionals may incorporate CSTM training into patient education to promote long-term self-management, improve adherence, and minimize recurrence of symptoms.

## 5. Conclusions

The findings of this study support the effectiveness and safety of an integrative self-care program combining cervical stabilization exercises with Thai self-massage for young adults with CNNP. Within this population, the CSTM program was associated with reductions in pain and disability and encouraged active participation in self-management. This approach may be incorporated into community- or home-based rehabilitation programs as a cost-effective and accessible self-care strategy. However, caution should be exercised when extrapolating these findings to older adults or individuals with more severe neck-related disability, and further studies are warranted to confirm its applicability across broader and more clinically diverse populations.

## Figures and Tables

**Table 1:** Baseline Participant Characteristics.

Characteristic	Randomized (*n* = 46)	
	CSTM (*n* = 23)	Control (*n* = 23)
Age (years), Mean (SD)	21.43 (1.75)	21.57 (1.20)
Gender; number of females (%)	20 (86.95)	17 (73.91)
Weight (kg), Mean (SD)	66.74 (17.77)	62.77 (14.64)
Height (m), Mean (SD)	1.65 (0.11)	1.61 (0.58)
BMI (kg/m^2^)	24.28 (5.90)	23.95 (5.25)

CSTM = Combination of cervical stabilization exercises and Thai self-massage, SD = Standard deviation.

**Table 2:** Within-Group Comparisons of Outcome Measures Over Time (Repeated Measures ANOVA).

Outcome	Groups	Baseline	Week 4 (Post-Test 1)	Week 6 (Post-Test 2)
Pain intensity: Mean ± SD	CSTM	4.96 ± 1.33	2.52 ± 1.34 *	1.65 ± 1.43 *
	Control	4.91 ± 1.16	3.61 ± 1.72 *	2.83 ± 1.70 *
Pressure pain threshold: Mean ± SD	CSTM	5.05 ± 1.43	7.33 ± 2.24 *	7.45 ± 2.80 *
	Control	5.73 ± 2.24	7.34 ± 2.66 *	7.20 ± 3.01 *
Neck Disability Index: Mean ± SD	CSTM	9.26 ± 2.97	4.74 ± 2.99 *	3.70 ± 2.48 *
	Control	9.91 ± 3.99	7.43 ± 4.10 *	6.00 ± 3.44 *

Note: CSTM = Combination of cervical stabilization exercises and Thai self-massage, SD = Standard deviation, * Significant improvement from baseline levels (*p* < 0.05).

**Table 3:** Adjusted Between-Group Comparisons of Post-Test Means Using ANCOVA.

Outcome	Week 4 (Post-Test 1)			Week 6 (Post-Test 2)		
	CSTM Mean (95% CI)	Control Mean (95% CI)	Difference (95% CI)	CSTM Mean (95% CI)	Control Mean (95% CI)	Difference (95% CI)
Pain intensity	2.59 (2.15–3.04)	3.53 (3.09–3.98)	0.94 * (0.31–1.58)	1.73 (1.16–2.31)	2.74 (2.17–3.32)	1.01 * (0.19–1.83)
Pressure pain threshold:	7.58 (6.80–8.37)	7.09 (6.31–7.88)	−0.49 (−1.62–0.64)	7.63 (6.65–8.61)	7.03 (6.05–8.01)	−0.59 (−2.01–0.82)
Neck Disability Index	4.83 (3.42–6.24)	7.34 (5.93–8.75)	2.51 * (0.49–4.54)	3.73 (2.50–4.97)	5.96 (4.73–7.20)	2.23 * (0.46–4.00)

Note: CSTM = Combination of cervical stabilization exercise and Thai self-massage, SD = Standard deviation, CI = Confidence interval, * Significant difference between groups (*p* < 0.05).

## Data Availability

Due to the inclusion of sensitive participant data, the datasets supporting these findings are not publicly archived. Access may be granted from the corresponding author for research purposes, following a formal request and ethical approval.

## Abbreviations

The following abbreviations are used in this manuscript:

CNNP	Chronic nonspecific neck pain
CSTM	A combined program of cervical stabilization exercises and Thai self-massage
CSE	Cervical stabilization exercises
